# Silibinin Effect on Methotrexate-Induced Hepatotoxicity in Rats

**DOI:** 10.5152/eurasianjmed.2022.20371

**Published:** 2022-10-01

**Authors:** Emine Yanaşoğlu, Mustafa Büyükavcı, Ayhan Çetinkaya, Gupse Turan, Mehmet Köroğlu, Hayrullah Yazar, Mehmet Emin Büyükokuroğlu

**Affiliations:** 1 Department of Pediatrics, Sakarya University Faculty of Medicine, Sakarya, Turkey; 2 Department of Pediatric Hematology and Oncology, Sakarya University Faculty of Medicine, Sakarya, Turkey; 3 Department of Physiology, Abant İzzet Baysal University Faculty of Medicine, Bolu, Turkey; 4 Department of Pathology, Sakarya University Faculty of Medicine, Sakarya, Turkey; 5 Department of Microbiology, Sakarya University Faculty of Medicine, Sakarya, Turkey; 6 Department of Biochemistry, Sakarya University Faculty of Medicine, Sakarya, Turkey; 7 Department of Pharmacology, Sakarya University Faculty of Medicine, Sakarya, Turkey

**Keywords:** Methotrexate, hepatotoxicity, silibinin, oxidative stress, thiol/disulfide

## Abstract

**Objective:**

Hepatotoxicity is one of the major side effects of methotrexate and limits its use. In this study, we investigated the hepatoprotective effect of silibinin and the role of oxidative stress markers and cytokines on high-dose methotrexate-induced hepatotoxicity in rats.

**Materials and Methods:**

In this study, rats were randomly divided into 5 groups (n = 7). Methotrexate (20 mg/kg, intraperitoneally) was administered on the first day in all groups except control. Silibinin was injected for 5 days to methotrexate–silibinin25, methotrexate–silibinin50, and methotrexate–silibinin100 groups at a dose of 25, 50, and 100 mg/kg/day, respectively. On the sixth day, blood and liver samples were obtained and rats were sacrificed. Serum total antioxidant capacity, total oxidant status, total thiol, native thiol, alanine aminotransferase, aspartate transaminase, bilirubin, albumin, tumor necrosis factor-alpha, and interleukin-10 levels were measured. In addition, a histopathological evaluation of liver tissues was performed.

**Results:**

Methotrexate reduced total antioxidant capacity and increased disulfide/total thiol ratio. Histopathologic examination revealed that methotrexate increased hepatic damage and 50 mg/kg/dose of silibinin prevented inflammatory cell infiltration in particular.

**Conclusion:**

Our results suggest that silibinin (50 mg/kg/day) may reduce the hepatic damage in methotrexate-induced hepatotoxicity in rats by increasing antioxidant capacity.

**Main Points**

- Hepatoprotective effect of silibinin (SLB) is well known. However, SLB effects and its mechanism on methotrexate (MTX)-induced liver injury in rats have not been studied so far.
- In this study, we demonstrated that 50 mg/kg/day of SLB reduced the MTX-induced hepatic damage in rats.
- The hepatoprotective effect of SLB is associated with antioxidant mechanisms.

## Introduction

Methotrexate (MTX) is an anti-neoplastic and anti-inflammatory agent used in the treatment of many autoimmune and malignant diseases. It is metabolized in the liver and has obvious cytotoxic effects on the liver, gastrointestinal tract, and bone marrow besides tumor cells.^1^ High dose of MTX therapy can lead to an increase in liver enzymes and histopathological changes in liver tissue. Methotrexate increases the serum level of tumor necrosis factor-alpha (TNF-𝛼) and alters the oxidant–antioxidant balance, which can be prevented by the administration of antioxidant agents.^2,3^ In the literature, there are studies investigating the effects of agents including infliximab, amifostine, ascorbic acid, and resveratrol on MTX-induced hepatotoxicity.^3-5^

Silibinin (SLB), the active extract of *Silybum marianum*, is an antioxidant, anti-inflammatory, anti-carcinogenic, anti-fibrotic, and hepatoprotective agent. It protects the liver from oxidative stress and inflammatory processes via the inhibition of reactive oxygen species (ROS) and secondary cytokines, leukotriene formation by Kupffer cells, chelation of metal ions, increase of ribosomal protein synthesis, and inhibition of neutrophil migration.^6-11^ In addition, SLB prevents hepatic fibrogenesis by reducing the pro-fibrogenic potential of hepatic stellate cells.^12^

Silibinin protects the liver tissue against toxicity induced by 𝛼-amanitin, itraconazole, and doxorubicin.^13-15^ It also attenuates the MTX-induced pulmonary injury and nephrotoxicity in rats.^16,17^ In this study, we investigated the effect and mechanism of action of SLB on MTX-induced liver injury in rats. We used the liver function tests and histopathologic evaluation for the investigation of hepatic injury. For investigating the role of oxidative stress, we determined the total antioxidant capacity (TAC), total oxidant status (TOS), and thiol/disulfide homeostasis. We measured serum levels of TNF-𝛼 and interleukin-10 (IL-10) to investigate the role of inflammatory cytokines.

## Materials and Methods

The study was carried on according to the Basic and Clinical Pharmacology and Toxicology policy for experimental and clinical studies.^18^ The study protocol was approved by the local ethics committee of Abant İzzet Baysal University, Experimental Animal Application and Research Center date: May 10, 2017, reference number: 2017/24. Written informed consent was obtained from all participants who participated in this study.

### Animal Model

The study was carried out using 35 female Wistar-Albino rats aged 8-12 weeks and weighing 200-250 g, at the Experimental Animal Application and Research Center of Abant İzzet Baysal University Rats were fed ad libitum water and standard pellets in 19-21^o^C of room temperature and 50-55 relative humidity.

### Experimental Design

The rats were randomly divided into 5 groups. Each group consisted of 7 animals and was treated as follows:

Group 1 (control): rats received nothing (sham control);

Group 2 (MTX): rats received intraperitoneal MTX (20 mg/kg, single dose) on the first day and dimethyl sulfoxide (DMSO) (0.5 mL/day) for 5 days;

Group 3 (MTX-SLB25): rats received intraperitoneal MTX (20 mg/kg, single dose) on the first day and SLB (25 mg/kg/day) for 5 days;

Group 4 (MTX-SLB50): rats received intraperitoneal MTX (20 mg/kg, single dose) on the first day and SLB (50 mg/kg/day) for 5 days;

Group 5 (MTX-SLB100): rats received intraperitoneal MTX (20 mg/kg, single dose) on the first day and SLB (100 mg/kg/day) for 5 days.

At the end of the experiment, the rats were sacrificed after withdrawing 4 mL of blood by cardiac puncture under xylazine (10 mg/kg)–ketamine (90 mg/kg) anesthesia. The liver tissues removed following sacrification were fixed with 10% neutralized formalin solution. Blood was collected in sterile tubes. Twenty minutes after collection, blood was centrifuged at 1500 rpm for 10 minutes. Serum obtained after the procedure was stored at –80°C until use.

### Drug and Chemicals

Silibinin was bought from Sigma (Sigma–Aldrich, St. Louis, Mo, USA) and dissolved in 5% DMSO. Methotrexate (Methotrexate Ebewe, 50 mg/5 mL, Sandoz, TR) and SLB or DMSO (0.5 mL) were administered at 1 hour intervals. To assess the dose-dependent effect of SLB, 3 different doses (25, 50, and 100 mg/kg/day) of SLB were used.

### Total Antioxidant Capacity and Total Oxidant Status

An automated analyzer (Beckman Coulter AU 680, Tokyo, Japan) and Rel Assay Diagnostics kits were used for the analysis. A novel spectrophotometric method, which was described previously by Erel, was used for the determination of TAC and TOS.^19,20^

Serum TAC and TOS levels were presented as millimole Trolox equivalent/L and H_2_O_2_ equivalent/L, respectively.

Oxidative stress index (OSI) was calculated as follows and presented as arbitrary units (AU):

OSI (arbitrary unit) = TOS (mmol H_2_O_2_ equivalent/L)/TAC (mmol Trolox equivalent/L).

### Total Thiol, Native Thiol, and Disulfide

Total thiol and native thiol levels were determined using an automated analyzer (Beckman Coulter AU 680) and Rel Assay Diagnostics kits, and the results were presented as micromole per liter. We used a previously described spectrophotometric method for the determination of thiol/disulfide homeostasis parameters.^21^

The disulfide level was calculated by dividing the difference between the total thiol and native thiol by 2. We calculated the disulfide/native thiol, disulfide/total thiol, and native thiol/total thiol ratios after achieving the disulfide, native thiol, and total thiol levels.

### TNF-α and IL-10

An automated micro-ELISA analyzer (Grifols, Triturus, Spain) and rat-specific kits (Thermo Fisher Scientific Rat TNF alpha and IL-10 Platinum ELISA Kit, USA) were used for the analysis. The results were presented as picograms per milliliter.

### Biochemical Markers

Serum aspartate aminotransferase (AST), alanine aminotransferase (ALT), alkaline phosphatase (ALP), lactate dehydrogenase (LDH) (U/L), total-direct bilirubin (mg/dL), and albumin (g/dL) levels were determined with an automated analyzer (Beckman Coulter AU 5800)

### Histopathologic Evaluation

Tissue samples taken from the liver were fixed with 10% formaldehyde and embedded in paraffin for light microscopic investigations. Tissue sections were stained with hematoxylin and eosin and were examined under a photomicroscope. Sinusoidal dilatation, inflammatory cellular infiltration, hepatocellular vacuolation, and necrosis were scored as absent (−), mild (+), moderate (++), and severe (+++). In addition, the total score was calculated for each case.

### Statistical Analysis

Descriptive statistics were used to compare the general features of all participants. The Shapiro–Wilk test was employed to compare the distributions of numerical variables. We calculated means ± standard deviation of variables that were normally distributed and medians (with minima and maxima) of those that were not normally distributed. The Student’s *t*-test, Mann–Whitney *U* test, analysis of variance, and Kruskal–Wallis H-test were used as appropriate to compare data between groups. IBM SPSS Statistics for Windows Software v.20 (IBM Corp., Armonk, NY, USA) was used for all statistical analyses. A *P*-value <.05 was considered to reflect statistical significance.

## Results

### Oxidative Stress Parameters

There were no differences between groups regarding TOS and OSI levels. Methotrexate exposure and SLB treatment had no effect on TOS and OSI. Total antioxidant capacity significantly decreased in MTX and MTX-SLB25 groups (*P *< .05); however, there was no decrease in MTX-SLB50 and MTX-SLB100 groups. Although there was no significant difference compared to MTX and control groups, reduced TAC was increased by SLB at the dose of 50-100 mg/kg (Table 1).

Total thiol and native thiol levels were significantly reduced in MTX group as expected (*P* < .05). Total thiol and native thiol levels rose in SLB-treated groups; however, the difference was not significant. Disulfide levels were significantly lower (*P *< .05) in MTX-SLB25, MTX-SLB50, and MTX-SLB100 groups. Disulfide/total thiol values were higher (*P *< .05) in MTX group compared to controls (Table 2).

### TNF-𝛼 and IL-10

Tumor necrosis factor-alpha and interleukin-10 levels in all groups were similar to the control group (Table 3). Methotrexate or silibinin exposure appeared to have no effect on either TNF-𝛼 or IL-10.

### Biochemical Markers

Alanine aminotransferase, alkaline phosphatase, and albumin levels were significantly lower in MTX-treated groups (*P* < .05). Lactate dehydrogenase levels were also lower in MTX-treated groups except for MTX-SLB50 (Table 4).

### Histopathologic Evaluation

**Figure 1.**
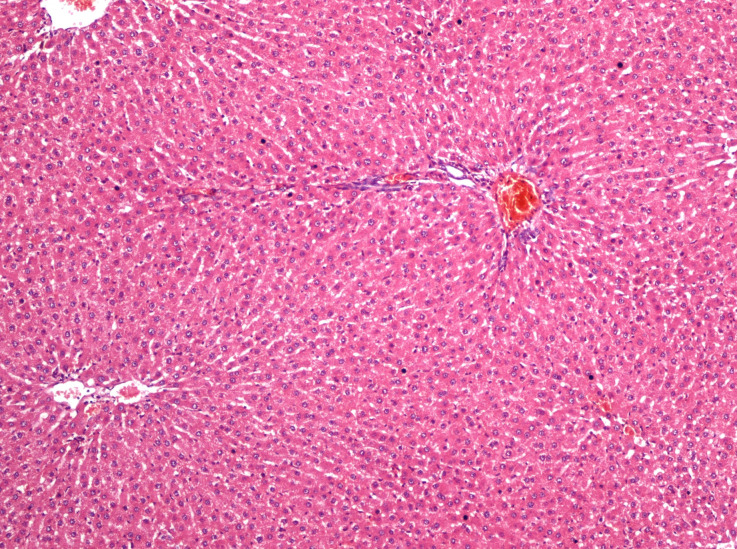
Photomicrograph of liver section of control group showing hepatocytes between the central vein and portal areas. (H&E ×100)

**Figure 2.**
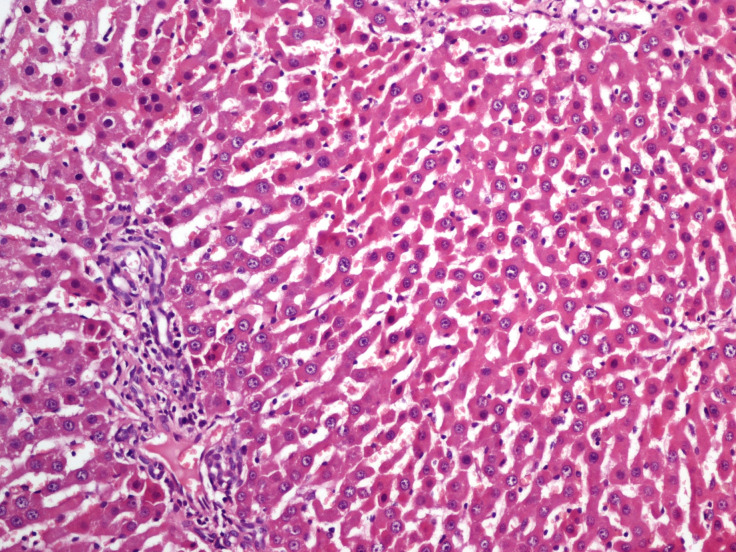
Photomicrograph of liver section showing the infiltration of mononuclear cells at portal area and dilated hepatic sinusoids. (H&E ×200)

**Figure 3.**
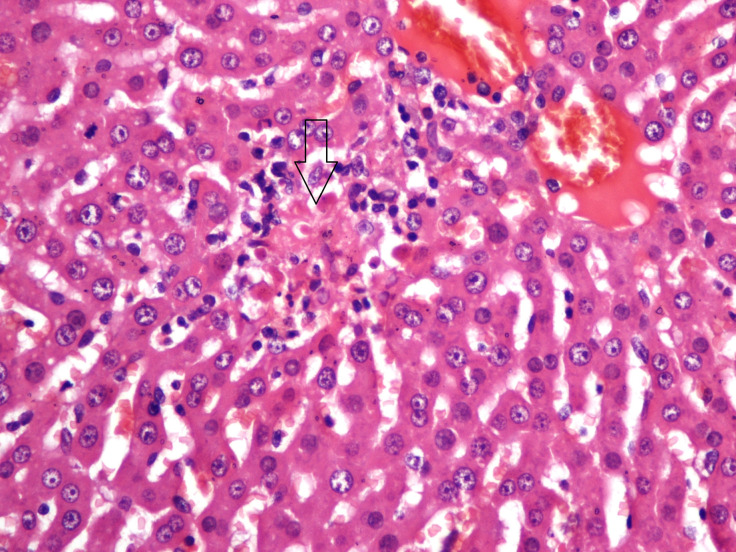
Photomicrograph of liver section showing parenchymal necrosis and markedly dilated hepatic sinusoids. (H&E ×400)

The sections of the control group demonstrated the normal structure of liver tissue (Figure 1). Methotrexate administration resulted in sinusoidal dilatation, inflammatory cell infiltration, hepatocellular vacuolation, or necrosis (Figures 2 and 3). Sinusoidal dilatation scores were higher in all MTX-induced groups compared to the control (*P* < .05).

Infiltration of inflammatory cells was prominent in MTX and MTX-SLB25 groups (*P* < .05). However, MTX-SLB50 group, similar to control group, demonstrated significantly lower infiltration than MTX and MTX-SLB25 (*P* < .05). Methotrexate-silibinin100 group was not different from both the MTX and control groups.

Hepatocellular vacuolation scores were significantly higher in MTX, MTX-SLB25, MTX-SLB50, and MTX-SLB100 groups than the control (*P* < .05). There was no difference between the groups regarding necrosis scores although all groups depicted necrosis except control and MTX-SLB50 (*P* > .05).

Total histopathologic scores for liver injury were remarkably higher in all MTX-induced groups than control. However, MTX-SLB50 group had a significantly lower score than MTX-only group (*P *< .05) (Table 5).

## Discussion

This study aimed to investigate the role of oxidative stress and inflammatory cytokines and the potential protective role of SLB, an antioxidant and hepatoprotective agent, in MTX-induced hepatic injury.

Antioxidant effects of SLB are shown in previous studies using the serum and tissue levels of superoxide dismutase, glutathione peroxidase, myeloperoxidase, and nitric oxide.^16,22,23^ Total antioxidant capacity, TOS, and thiol/disulfide homeostasis are new oxidative stress markers. We hypothesized that SLB, as an antioxidant scavenger, reverses the MTX-induced decline in serum TAC levels and a rise in TOS and OSI levels.

It has been reported that MTX has different effects on TAC and TOS. Akbulut et al^3^ reported that MTX (20 mg/kg) exposure did not affect serum TAC, however, increased TOS levels in rats. Selimoglu et al^24^ reported that the same dose of MTX declined the levels of TAC and raised the levels of TOS and OSI in serum and tissue. They also exerted that antioxidant carvacrol and pomegranate extract increased the TAC and decreased the OSI. In the present study, TAC significantly decreased in MTX and MTX-SLB25 groups; however, there was no difference in MTX-SLB50 and MTX-SLB100 groups compared to control. It may be attributed to positive effect of SLB on antioxidant capacity at a dose of 50-100 mg/kg. However, TOS and OSI levels were similar to controls in all groups.

The results about thiol/disulfide homeostasis also confirmed the antioxidant effect of SLB. Total thiol, native thiol, and native/total thiol levels decreased while disulfide/total thiol levels increased in MTX group. It was not observed in SLB-treated groups and was attributed to antioxidant effect of SLB on MTX-induced oxidative stress (especially increased disulfide/total thiol ratio). The lower disulfide levels in MTX-SLB groups than control also confirmed this interpretation. The association between high-dose MTX and thiol/disulfide homeostasis was reported for the first time in the literature.

Both MTX and SLB affect inflammation through the modulation of TNF-𝛼 and IL-10 levels.^5,25^ Methotrexate reduces the secretion of proinflammatory cytokines and TNF-𝛼 at pharmacological doses but increases at higher doses.^2,26^ Previous studies have demonstrated that 20 mg/kg of MTX (single dose) increases the proinflammatory cytokines after 20 days of administration and 7 mg/kg/day of MTX (3 days) increases the oxidative stress, and ROS 24 hours after the end of treatment.^27,28^ However, high dose of MTX had no effect on the proinflammatory cytokine TNF-𝛼 and the anti-inflammatory cytokine IL10 in this study.

In the literature, there are many studies exerting the anti-inflammatory effect of SLB and derivatives. Silymarin potentiates the anti-inflammatory effects of celecoxib by reducing the TNF-𝛼 and ROS in rats with osteoarthritis.^29^ Silibinin decreases the serum TNF-𝛼 levels and increases IL-10 compared to placebo in patients with active rheumatoid arthritis treated with low-dose MTX.^30^ Schümann et al^31^ reported that SLB has hepatoprotective effect by inhibiting the intrahepatic expression of TNF and increasing the synthesis of IL-10 in rats with T cell-dependent liver injury. Unlike the mentioned studies, we did not observe a difference in the TNF-𝛼 and IL-10 levels between the SLB-treated groups and the control group.

At the beginning of the research, we hypothesized that MTX would cause an increase in liver function tests. However, MTX therapy paradoxically decreased the serum ALT and ALP levels in all groups, while the AST and bilirubin were similar compared to controls. There are different results reported in the literature regarding this issue. Dalaklıoğlu et al^28^ reported that 3 days of MTX (7 mg/kg/day) therapy results in a remarkable increase in serum AST, ALT, and ALP levels. Akbulut et al^3^ detected a significant decrease in serum AST and ALP levels and a significant increase in ALT levels following 20 mg/kg of MTX infusion. Antioxidants were found to be effective in the prevention of hepatotoxicity in both studies. Massive hepatic necrosis may also lead to the reduction of liver enzymes.^32^ However, the histopathologic evaluation revealed only mild necrosis in our study.

Long-term (28 days) SLB treatment at a dose of 5.1 mg/kg/day declines the serum AST levels in children with grade 2 hepatotoxicity associated with the maintenance therapy of acute lymphoblastic leukemia.^33^ Hagag et al^34^ reported that 1-week silymarin treatment following MTX administration attenuates the MTX-induced hepatotoxicity by reducing the serum AST, ALT, and ALP levels in children with acute lymphoblastic leukemia. Silymarin also reduces the serum AST, ALT, ALP, and bilirubin levels and prevents MTX-induced liver dysfunction in rats.^35^ In the present study, 3 different doses of SLB had no effect on ALT, ALP, and bilirubin levels.

Silibinin administration prevents the increase of ALT and AST due to MTX therapy in rats.^16^ Unlike our work, SLB has been given for 7 days preceding MTX infusion in the mentioned study. It can be attributed to the cytoprotective effect of SLB from toxic exposure before the cellular injury. We administered the MTX treatment first, considering that the same effect may also apply to cancer cells.

Albumin is synthesized by the liver and is an important indicator of liver function. The serum levels reflect its rate of synthesis, degradation, or clearance. The depressed serum levels are not specific to liver disease and may also arise in renal diseases such as nephrotic syndrome.^36^ In this study, serum albumin levels decreased in MTX, MTX-SLB25, MTX-SLB50, and MTX-SLB100 groups compared to control. It cannot be attributed to hepatic dysfunction because of the long half-life of albumin in serum as long as 20 days. The serum albumin levels are usually normal in acute diseases like drug-related hepatotoxicity.^36^ Renal losses may also be a contributing factor because of the nephrotoxic side effects of MTX.^37^ However, nephrotoxicity was not evaluated in this study. Furthermore, albumin has a significant role in extracellular antioxidant defense in blood plasma. Albumin supplementation improves extracellular thiol-dependent anti-oxidant protection in critically ill patients.^38,39^ We observed the nadir albumin levels in MTX and MTX-SLB25 groups having the least TAC levels. The above findings imply that the serum level is decreased because of the use of albumin in the antioxidant defense mechanism.

Akbulut et al^3^ reported that antioxidant agents alleviate the MTX-induced structural and functional hepatic damage in rats. Extracts of milk thistle also can prevent MTX-induced liver dysfunction and fibrosis in rats.^35^ Histopathologic examination revealed that high-dose MTX infusion causes hepatic damage in the form of sinusoidal dilatation, inflammatory cell infiltration, hepatocellular vacuolation, or necrosis in this study. In particular, inflammatory cell infiltration increased in MTX-only group and improved in MTX-SLB50 group. Total histologic scores were also significantly higher in MTX-treated groups than control. However, it was significantly lower in MTX-SLB50 group than MTX-only group but not in MTX-SLB25 and MTX-SLB100. This shows that increasing the dose does not increase the anti-inflammatory effect of SLB.

In conclusion, SLB (50 mg/kg/day) reduced the hepatic damage in MTX-induced hepatotoxicity in rats. However, this effect did not reflect on biochemical markers. The hepatoprotective action of SLB was not through cytokines but through antioxidant effects.

The limitations of our study were given as follows:

1. This study also aimed to evaluate the SLB effect on serum MTX levels. However, we had no diagnostic kits for the measurement of serum MTX levels and we could not do it.

2. We could not evaluate the nephrotoxicity caused by MTX and therefore the role of nephrotoxicity on serum albumin levels.

## Figures and Tables

**Table 1.** Serum TAC, TOS, and OSI levels (Mean ± SD)

	Control	MTX	MTX-SLB25	MTX-SLB50	MTX-SLB100
TAC	1.37 ± 0.04	0.88 ± 0.14^a^	0.9 ± 0.3^a^	1.02 ± 0.27	0.97 ± 0.39
TOS	6.7 ± 6.04	11.7 ± 10.3	37.2 ± 44.60	48.5 ± 76.64	39.9 ± 63.6
OSI	4.9 ± 4.3	13.7 ± 12.3	34.1 ± 34.7	36.2 ± 48	30.9 ± 37.3

^a^ *P* < .05 compared to control.

TAC, total antioxidant capacity (mmol Trolox Equivalent/L); TOS, total oxidant status (H_2_O_2_ Equivalent/L); OSI, oxidative stress index (arbitrary unit); SD, standard deviation.

**Table 2.** Serum TT, NT, Disulfide, D/N, D/T, and N/T Levels (Mean ± SD)

	Control	MTX	MTX-SLB25	MTX-SLB50	MTX-SLB100
TT (µmol/L)	2189.3 ± 193.3	1259 ± 309.7^a^	1549.8 ± 1320	1948.6 ± 1355.1	1685.2 ± 1600
				
NT (µmol/L)	884 ± 171	311 ± 93^a^	892 ± 1233	1005 ± 1622	955 ± 1494
				
Disulfide (µmol/L)	651 ± 24.1	473.5 ± 127.4	328.6 ± 89.1^a^	471.2 ± 153.9^a^	364.6 ± 108,2^a^
				
D/N (%)	75 ± 12	156 ± 48	190 ± 264	148 ± 106	105 ± 75
				
D/T (%)	29 ± 2	37 ± 2^a^	28 ± 14	31 ± 13	29 ± 12
				
N/T (%)	40 ± 4	25 ± 5^a^	41 ± 28	35 ± 27	40 ± 23

^a^ *P* < .05 compared to control.

TT, total thiol; NT, native thiol; D/N, disulfide/native thiol; D/T, disulfide/total thiol; N/T, native/total thiol; MTX, methotrexate; SLB, silibinin.

**Table 3.** Serum TNF-𝛼 and IL-10 Levels (Mean ± SD)

	Control	MTX	MTX-SLB25	MTX-SLB50	MTX-SLB100
TNF-𝛼 (pq/mL)	23.16 ± 3.80	22.35 ± 2.42	25.35 ± 6.62	23.59 ± 2.63	29.62 ± 6.07
IL-10 (pg/mL)	128 ± 63	160 ± 81	289 ± 174	113 ± 57	235 ± 170

TNF-𝛼, tumor necrosis factor-alpha; IL, interleukin; MTX, methotrexate; SLB, silibinin; SD, standard deviation.

**Table 4.** Serum AST, ALT, ALP, LDH, Total Bilirubin, Direct Bilirubin, and Albumin Levels (Mean ± SD)

	Control	MTX	MTX-SLB25	MTX-SLB50	MTX-SLB100
AST (U/L)	137.9 ± 15.4	114.8 ± 29.1	101 ± 30.7	116 ± 27.8	125.6 ± 76.9
ALT (U/L)	67.5 ± 10.2	28 ± 3.9^a^	26.3 ± 8.8^a^	33.9 ± 9.8^a^	30.6 ± 19.4^a^
ALP (U/L)	242.2 ± 47.4	69.67 ± 71.5^a^	55 ± 13.2^a^	38 ± 17.5^a^	49.4 ± 31.8^a^
LDH (U/L)	843 ± 198	416 ± 152^a^	280 ± 167^a^	569 ± 284	331 ± 352^a^
Total Bilirubin (mg/dL)	0.16 ± 0.01	0.13 ± 0.03	0.15 ± 0.05	0.16 ± 0.09	0.17 ± 0.06
Direct Bilirubin (mg/dL)	0.027 ± 0.01	0.026 ± 0.01	0.036 ± 0.02	0.022 ± 0.01	0.046 ± 0.02
Albumin (g/dL)	3.15 ± 0.1	2.17 ± 0.3^a^	2.09 ± 0.4^a^	2.46 ± 0.2^a^	2.32 ± 0.3^a^

^a^ *P* < .05 compared to control.

AST, aspartate aminotransferase; ALT, alanine aminotransferase; ALP, alkaline phosphatase; LDH, lactate dehydrogenase; MTX, methotrexate; SLB, silibinin; SD, standard deviation.

**Table 5.** Sinusoidal Dilatation (SD), Inflammatory Cellular Infiltration (ICI), Hepatocellular Vacuolation (HV), and Necrosis Scores [Median (Min-Max)]

	Control	MTX	MTX-SLB25	MTX-SLB50	MTX-SLB100
SD	0 (0-0)	2 (1-2)^a^	1.5 (1-2)^a^	1 (1-2)^a^	1 (1-2)^a^
ICI	0 (0-0)	1 (0-1)^a^	1 (0-2)^a^	0 (0-0)^b,c^	0 (0-1)
HV	0 (0-0)	1 (0-1)^a^	1 (1-1)^a^	1 (1-1)^a^	1 (1-2)^a^
Necrosis	0 (0-0)	0 (0-1)	0 (0-1)	0 (0-0)	0 (0-1)
Total	0 (0-0)	4 (3-4)^a^	3 (2-6)^a^	2 (2-3)^a,b^	3 (2-4)^a^

^a^P < .05 compared to control, ^b^ *P* < .05 compared to MTX group, ^c^ *P* < .05 compared to MTX-SLB25 group.

SD, sinusoidal dilatation; ICI, inflammatory cellular infiltration; HV, hepatocellular vacuolation; MTX, methotrexate; SLB, silibinin.
